# Addictive Features of Social Media/Messenger Platforms and Freemium Games against the Background of Psychological and Economic Theories

**DOI:** 10.3390/ijerph16142612

**Published:** 2019-07-23

**Authors:** Christian Montag, Bernd Lachmann, Marc Herrlich, Katharina Zweig

**Affiliations:** 1 Department of Molecular Psychology, Institute of Psychology and Education, Ulm University, 89081 Ulm, Germany; 2 Serious Games Engineering, TU Kaiserslautern, 67663 Kaiserslautern, Germany; 3 Algorithm Accountability Lab, TU Kaiserslautern, 67663 Kaiserslautern, Germany

**Keywords:** social media/messenger apps, Facebook, WhatsApp, Internet addiction, smartphone addiction, Internet use disorder, smartphone use disorder

## Abstract

Currently about 2.71 billion humans use a smartphone worldwide. Although smartphone technology has brought many advances, a growing number of scientists discuss potential detrimental effects due to excessive smartphone use. Of importance, the likely culprit to understand over-usage is not the smartphone itself, but the excessive use of applications installed on smartphones. As the current business model of many app-developers foresees an exchange of personal data for allowance to use an app, it is not surprising that many design elements can be found in social media apps and Freemium games prolonging app usage. It is the aim of the present work to analyze several prominent smartphone apps to carve out such elements. As a result of the analysis, a total of six different mechanisms are highlighted to illustrate the prevailing business model in smartphone app development. First, these app-elements are described and second linked to classic psychological/economic theories such as the mere-exposure effect, endowment effect, and Zeigarnik effect, but also to psychological mechanisms triggering social comparison. It is concluded that many of the here presented app-elements on smartphones are able to prolong usage time, but it is very hard to understand such an effect on the level of a single element. A systematic analysis would require insights into app data usually only being available for the app-designers, but not for independent scientists. Nevertheless, the present work supports the notion that it is time to critically reflect on the prevailing business model of ‘user data in exchange for app-use allowance’. Instead of using a service in exchange for data, it ultimately might be better to ban or regulate certain design elements in apps to come up with less addictive products. Instead, users could pay a reasonable fee for an app service.

## 1. Introduction

According to current estimates, about 2.71 billion humans use a smartphone worldwide [1]. Without doubt, one of the major driving forces towards a totally connected world with the Internet available in everyone’s pocket was the inception of the iPhone in 2007 [2]. The wide distribution of smartphones led to unprecedented opportunities enabling humans to find their way in unknown territory via services such as Google Maps, to reach out to friends and business partners via a myriad of communication channels, and abundant easy access to knowledge earlier only available via visiting a library and the study of lexicons.

### 1.1. Perils of Smartphone Use

Despite the many advantages of the smartphone, it is heavily discussed among scientists around the globe if problematic usage of the smartphone could have detrimental effects on our mental health [3,4]. Furthermore, associations between anxiety disorder, depression, and problematic use of the smartphone have been observed (underlining the sincerity of this topic), but the causal relationship of effects between the constructs is still unclear [5]. In any case, new research suggests that the many interruptions due to high frequency use and the many daily incoming messages fragmenting everyday life could reduce productivity at work [6,7,8] and lower a person’s well-being [9,10]. Another research group stresses that the availability of smartphones even might lead to “brain drain”, when the device is present on the desk while working on a fluid intelligence task or working memory task [11]. In this context, a recent review summarized the literature on cognitive functions [12]. Therefore, this is area of research is not further presented in detail, here.

Beyond the mentioned issues, (excessive use of) smartphones is known to impact on social communication in terms of reducing smiles when interacting with strangers [13], enjoyment of face-to-face interaction [14] or drawing parent’s attention away from their children [15]. This might result in a loss of empathy, although so far only correlations between excessive Internet use and lower empathy/social skills could be established [16,17], with only weak associations between excessive smartphone usage and empathy [18]. Again, note that the latter associations base on correlations and so far it is not clear what is cause and what is effect. Moreover, important insights of a study are mentioned which on the one hand observed reduced social skills in excessive Internet users, but on the other hand observed enhanced empathy when the Internet is used in a healthy way [19].

Beyond these observations, a growing number of scientists are focusing on the question of whether excessive smartphone usage could resemble a full-blown addiction [20,21,22]. Of importance, ‘smartphone addiction’ is currently not recognized in the official diagnostic manuals such as the International Classification of Diseases-11th Revision (ICD-11) issued by the World Health Organization (WHO) or the Diagnostic and Statistical Manual of Mental Disorders (DSM-5) issued by the American Psychiatric Association (APA). Both ICD-11 and DSM-5 offer practitioners and researchers alike valuable guidance in psycho-diagnostics. With respect to the general debate on the addictive nature of excessive usage of online content, it is noteworthy that Gaming Disorder, as a specific form of ‘Internet addiction’, has been included in ICD-11 [23]. This means that a first form of ‘Internet addiction’ is recognized as an official health condition, now.

Given the nomenclature of ‘Gaming Disorder’ in ICD-11, but also ‘Internet Gaming Disorder’ in DSM-5 (see Section 3 in the DSM-5’s appendix), many researchers currently prefer the term of ‘Internet Use Disorder’ (IUD) over the classic term ‘Internet addiction’ [24,25,26]. With this wording, scientists also stress that a certain kind of online usage and not the Internet per se poses a problem. Therefore, and in line with a prominent model to characterize and understand IUD by Brand et al. [26], the term IUD is also used in the following. Explicitly, it is mentioned that researchers should not over-pathologize everyday life activities [27] and aim at a unification of terms used in the literature in line with the officially suggested nomenclature.

In this context, one currently might prefer the term Smartphone Use Disorder (SUD) over ‘smartphone addiction’ and understand SUD best as a mobile form of IUD. Of note, many researchers currently use the term problematic smartphone use (PSU), which from our perspective is also ‘problematic’, because it is not clear if PSU represents a transit zone from healthy towards psychopathological behavior or the end of the spectrum. Correctly, it has been pointed out, that the smartphone does not represent the culprit to understand excessive use of this device [28,29]. Instead one must focus on the manifold installed applications on the smartphones to understand why people are getting ‘hooked’ to their devices [30]. In this context, it will be of relevance to also distinguish between social and non-social forms of smartphone use to ultimately understand its addictive potential. Interestingly, it has been shown in recent works that higher extraversion (going along with more assertiveness, but also sociality) is not linked to SUD [31,32]. Hence, core social use of social media apps on smartphones (e.g., to stay in touch with friends) might represent the healthy form of smartphone use, reflecting also homo sapiens’ urge for social bonding as also outlined in Pankseppian Affective Neuroscience Theory [33]. This all being said, it is understandable that the most prominent categories currently being researched—potentially causing addiction-like symptoms when being overused/or used in the wrong way—are social media/messenger platforms and Freemium games.

As prominent representatives of social media applications, Facebook, Snapchat, Instagram, and Twitter can be named and as a prominent messenger application WhatsApp or Signal. Without doubt, this is the Western view on this topic, whereas in Asia (particularly in China) the application WeChat dominates the market as a hybrid between social media and messenger application plus pay service [34].

The aforementioned term, Freemium, is a hybrid of the English words ‘free’ and ‘premium’. In the realm of Freemium games, this means that the basic version of a game can be downloaded at no monetary cost. However, the person playing a game pays either with his/her data produced while playing, by paying attention to ads, e.g., in exchange for further game-energy, or by paying money to access ‘premium’ content. Freemium games are very relevant for the present work investigating elements of apps built to prolong usage time, because such games most often run with the business model of ‘gaming service in exchange for your data’.

In a work by Montag et al. [35] it has been demonstrated that about 30% of daily smartphone use is accounted for by WhatsApp and Facebook usage in a German sample mainly ranging between ca. 15 and 35 years. Moreover, a recent work by Sha et al. [28] showed that the concepts of SUD and WhatsApp Use Disorder overlap in large parts (correlation of rho = 0.68). A new work underlines the overlap between SUD and excessive social media use, but also links between SUD and (Internet) Gaming Disorder [29]. Once more, this suggests that for the understanding of excessive smartphone use, researchers must also look at the high frequent use of specific applications on the smartphone and not only on the mere use of the total/frequent usage of the smartphone. The focus on social media/messenger applications alone would be a too narrow approach, because according to current numbers also Freemium games play a crucial role when one wants to understand what people are doing on their smartphones. To illustrate this: According to statista.com, “In early 2016 … U.S. gamers played an average of 3.6 mobile games per month, and 1.3 games on a daily basis” [36].

### 1.2. Main Research Questions in the Available Literature Investigated so Far

Looking at the growing literature dealing with Internet and Smartphone Use Disorder, it becomes apparent that most studies apply a confirmatory approach to understand IUD/SUD. Since the beginning of this research field with the case study of Kimberly Young [37] scientists tried to test if already existing symptoms in the realm of pathological gambling and/or substance related addictions can be transferred or applied to this potentially new form of a (online) behavioral addiction. It is not surprising that many existing self-report inventories in the field still test if symptoms such as preoccupation with the drug (here the Internet or a certain application), withdrawal symptoms, development of tolerance, loss of control, to name a few, can be associated with excessive Internet usage. Beyond such a confirmatory approach, many studies until this day deal with the important question if and what psychiatric co-morbidities can be observed when dealing with IUD and SUD. Of note, earlier works demonstrated co-morbidities with ADHD and depression [38,39,40], but also burnout [41] when investigating IUD. Earlier, links between SUD and depression/anxiety disorders have been already mentioned [5]. Of high importance, in the last years the research field has moved forward beyond these questions. Now, researchers also look at the processes explaining how addictive behaviors develop out of habits in the context of IUD and SUD. Up to now, presumably the most promising model to understand this process is the aforementioned I-PACE model by Brand et al. [26]. It argues that a complex interaction between person–affect–cognition and execution variables underlie the development of IUDs. IUDs are explicitly mentioned in plural here because from the introduction it should be clear that distinct forms exist such as Gaming Disorder or Internet Communication Disorder (ICD; or ‘social media addiction/use disorder’). This perhaps warrants a more focused research approach—instead of relying too broadly on IUD and SUD (see [31,32] and Montag et al. [42] for overlaps between generalized and specific forms of IUD). Of note, neuroscientific approaches have also been abundantly applied to understand the psycho-biological mechanisms underlying the different forms of IUD/SUD. Due to space restriction, it is referred to a new review study here [43].

Although, the different strains of research presented so far in Section 1 led to valuable insights into the nature of IUDs, only few studies until now aim to understand why specific online applications and their in-built elements are potentially addictive [44,45]. This should be achieved with Section 3 of this work. Of note, in the present work, the term ‘application(s)’ describes services such as Facebook or Instagram on platform level, whereas the term ‘elements/features’ refers to certain parts of the applications investigated further in Section 3. Furthermore, it is stated that the terms ‘elements’ and ‘features’ are used in an exchangeable way.

## 2. Methods

In the current work, several features of social media/messenger platforms and Freemium games are presented, which have been developed to grasp a maximum of the online user’s attention. The ‘classic’ app-business model of Silicon Valley asks the user not to pay with money to be able to use a certain application but with his/her personal data. Consequently, app developers have a keen interest in designing their online platforms with the goal to keep users occupied as long as possible [30]. This in turn means that more data per person can be harvested. With an increasing amount of data, revenues tend to rise, due to more effective microtargeting (i.e., sending a person customized ads). This is also shown by Matz et al. [46] demonstrating how clicks and buys can be boosted by the use of personalized ads. In the following, several app mechanisms are presented in alphabetic order. Some of these thoughts have been already covered in a chapter published by the authors in German language [47]. With the present paper, the main points of this earlier work should be made accessible for an international audience. Of importance, the present work is not a systemic review, but rather presents first insights into elements built-in social media and Freemium game apps. These insights have been derived by analysis of prominent and often used apps such as Facebook, WhatsApp, or Candy Crush. What follows is an overview of features built into social media/messenger and Freemium game applications likely prolonging app usage.

## 3. Results

In this section, we will cover six prominent psychological/economic mechanisms built-in social media apps and/or Freemium games. For an overview, see also Table 1.

### 3.1. Endless Scrolling/Streaming and the Concept of Flow

App and platform technologies are designed to be immersive, hence producing flow while using a certain app. Flow itself is a positive state of mind, which can promote high productivity. An important prerequisite for flow is that the difficulty of a task matches the ability of a person. If there is no fit, the person is either feeling anxious (the task is too difficult) or bored (the task is too easy). Other flow prerequisites are a clear goal set and a sense of personal control over the task. For a detailed introduction into the concept of flow and additional prerequisites, see the seminal work by Csikszentmihalyi [48].

Flow is needed and warranted in different kinds of work processes, however, it can actually be ‘fatal’ in the case of applications on the smartphone or other ‘ubiquitous’ devices, when attention should be focused somewhere else: It is well known that flow goes along with a feeling of time distortion and this is exactly what many developers of social media apps and Freemium games aim to achieve—a person being so immersed that he or she is forgetting about time and space while using a platform or app (cf., [30,49,50]). The distracting aspects of many applications on the smartphone are also a well-known danger for every day traffic [51] and in many countries led to the prohibition of smartphone use while driving a car or riding a bike.

One technique used to prolong usage time in this context is the endless scrolling/streaming feature. For example, on the video platform YouTube one can scroll down endlessly with apparently no end. By endlessly scrolling down, the user is getting more and more immersed (perhaps also on a motor level as perceiving the scrolling as a playful activity) while not coming to a natural stop, where he/she might easily reconsider to quit or change the platform. The behavior of endless scrolling is enhanced, because the user finds from time to time something rewarding (e.g., a funny or interesting video), hence intermittent conditioning principles are observed here (see also research on intermittent conditioning principles in the context of slot machine mechanisms by Harrigan & Dixon [52]). The same phenomenon is at work when ‘auto play’ is the default: e.g., when watching a video on YouTube or the favorite series on Netflix or Amazon Prime. As soon as one video is at the end, the next video begins with either a similar content (on YouTube) or the second episode of a TV show at Netflix and so forth. By this process, viewers get more and more absorbed, which makes it hard to stop watching.

### 3.2. Endowment Effect/Mere Exposure Effect

The endowment and the mere exposure effect play important roles in the areas of social media and games. One very prominent game including a simple variant of both effects is called “Hayday” which was released in 2012. This game is still very popular today but already belonged to the most successful Freemium games in 2013 [53]. In this game, the user aims at building a large farmland, where he/she is entitled to feed cattle, harvest crops and follows other agricultural related activities. As with other games, progress without investing money is very slow, which means that after some fast progress at the beginning of the game (to attract people to the game), rewards in terms of the urgently needed relevant game currency are scarce.

Where do the endowment effect and the mere exposure effect come in? The endowment effect describes that buying or owning a product leads to a higher (emotionally felt) value of the product, often way beyond its actual value [54,55]. In real life, this would explain why after buying a cheap souvenir for 2.99€, this thing starts to rise in the subjectively perceived value: reasons for this could be, that carrying the souvenir home feels like an investment and that fond memories of the holiday are also attached to it. This leads to the effect that the value attached to it, if, e.g., asked to sell the souvenir to someone else, often exceeds the original price. Both ownership and loss aversion are discussed as reasons for the endowment effect.

In the context of smartphone games, this plays out as follows: every time players visit the app platform and invest more time in the construction of the virtual world, it will get harder for them to detach from the game or even delete the app. This is in particular the case, if the progress has been slow and it took the users a very long time to construct the online world. It is their own little world now (resulting in the endowment effect). Also, of importance is the mere exposure effect [56]. The mere exposure effect describes that the more often you are exposed to a certain (neutral) thing or application (here a game), the more you like it. This has even been shown for the initials of your name (for an introduction in the initial preference task, e.g., see Sariyska et al. [57]) and seems to be applicable also for many of the gaming or social media apps.

### 3.3. Social Pressure

A successful way to increase data flow in an application is built into the popular messenger app WhatsApp. WhatsApp represents one of the most successful messenger services used worldwide to exchange text messages and pictures. At the time of writing, WhatsApp has about 1.5 billion users worldwide [58].

Users on WhatsApp are nudged (to encourage someone to do something in a way that is gentle rather than forceful or direct [59]) to communicate fast and often on WhatsApp due to the ‘double tick function’. If a user sends a message to a friend, the sender is presented with two gray ticks, which means that the message has successfully arrived at the recipient’s phone. If the recipient reads the message, the grey ticks turn blue. As both sides know about these rules, social pressure emerges. Both parties likely expect a fast answer, above all, if the message apparently has been read. This way, the users of the popular WhatsApp messenger are nudged towards faster communication via social pressure. This process is also known to undermine well-being [60] and might in parts be responsible for rising social concerns when people start to use smartphones [61]. Clearly also a link between features such as the double ticks and the concept of Fear of Missing Out (FoMO) can be established, with FoMO describing the anxiety/fear to miss something in one’s own social network [28,62].

Of note, the economic principle of ‘nudging’ can also be illustrated by the default settings of a fresh WhatsApp installation. The default mode of WhatsApp comes with the ‘double tick function’ being activated. Most users do not know that they are able to deactivate this app-feature. In general, the ‘power of defaults’ is a well-known principle applicable to decision making in many different areas and especially in software applications [63,64]. The percentage of users that actually take the time to check or even change the default settings may depend on the specific target group, the importance of the application, and the application design. For instance, there is an anecdotal report by Microsoft that only 5% of Word users change the defaults settings [65].

In sum, system design of apps nudges a person to behave in a certain direction. For person characteristics influencing the perception of these mechanisms, please see Mai et al. [66]. Social pressure plays not only a role on social media platforms to enhance user engagement, but also in computer games. Prominent games such as the massively multiplayer online-role play game (MMORPG) called World of Warcraft (WoW) enable participants to meet in so called ‘guilds’, hence groups of persons who meet at a certain time online to go on a mission together (in the aforementioned WoW game this could be a ‘raid’). As such a guild in particular performs well when being complete, the pressure on the individual of such an online group rises to be at a certain time in the online world to support one’s own guild. This goes along with a stronger focus in life on online activities instead on perhaps more pressing issues in the offline world.

### 3.4. Show Users of an App What They Like

A strong feature for prolonging usage time on social media platforms is the prominent ‘Newsfeed’ as built-in in the Facebook application. Over the years, Facebook developed machine learning algorithms studying the behavior of their users in detail on the platform. In order to get to know their users better, they do not only record what people ‘like’ (i.e., give a ‘thumbs up’) but also how long they hover over a certain post. This could be interpreted as showing a special interest in a certain area. ‘Textmining’ enables Facebook & Co. to do sentiment analysis and to understand not only what is interesting for their users but also in what mood they are [67,68,69]. Although, the role of mood for buying decisions/advertising is complex [70,71], such a variable might be of (high) interest to many of the companies behind manifold smartphone/Internet applications. The analysis of the different elements presented in this paragraph technically can all be used to plant the most interesting news in one’s own personalized ‘Newsfeed’.

The ‘Newsfeed’ on Facebook is the entry for every user when logging in. Facebook has a great interest in studying the behavior of each person at perfection and in much detail, so that at best only such information is presented in the ‘Newsfeed’ which is most interesting for the user. Otherwise, people could get bored and close the browser window. For a work dealing with user beliefs considering how the algorithm applied behind the ‘Newsfeed’ works, please see Rader & Gray [72].

### 3.5. Social Comparison and Social Reward

Perhaps one of the most prominent features of social reward mechanisms in social media is the iconic ‘thumbs up’ (giving or getting a ‘Like’; (co-)developed by Justin Rosenstein, former worker at Facebook; see also independent.co.uk [73]). Likes demonstrate either positive social feedback on one’s own posts or to give another person such a feedback. The power of such feedback has also been proven neuroscientifically, when Instagram users are confronted with their own posted pictures from their account which were manipulated by being presented either with many or few ‘Likes’ (in this case hearts [74]). Pictures being presented with many Likes elicit stronger activity in the ventral striatum, an area involved of the processing of a rewards [74,75]. It has even been demonstrated that lower gray matter volumes of the nucleus accumbens are associated with longer and higher frequent usage of the Facebook app on smartphones underlining its addictive power [76]. Additionally, several other studies observed that lower gray matter volumes were associated with higher addictive tendencies [77,78].

So far, in this paragraph only social reward via the ‘Like’ function has been discussed. Beyond that, processes of social comparison also play an important role for revisiting social media platforms because a person gets information on how he/she is perceived by their social network. Without doubt, the social comparison process is not unproblematic, because it might lead to lower self-esteem [79] and the size of one’s own real social network to be successfully handled seems to be limited by the so called Dunbar’s number (a theoretical limit to the number of people with whom any individual is able to sustain a stable or meaningful social relationship, usually considered to be roughly 150 [80]), likely also valid on social media channels such as Twitter [81]. Social comparison also plays a role in Freemium games, when one thinks of the highscore-boards where you can compare your scores in a game with those from others.

### 3.6. Zeĭgarnik/Ovsiankina Effect

Classic studies by Zeĭgarnik [82] and Rickers-Ovsiankina [83] made interesting observations concerning memory functions and actions taken after being interrupted while performing a task. In the classic work by Zeĭgarnik, participants of her study got interrupted while solving a puzzle and, in the aftermath, best remembered those tasks where they were interrupted compared to those tasks which they successfully could end. From the same work group, Rickers-Ovsiankina [83] then observed that persons not only seem to be better at remembering the tasks where they got interrupted, but several of the experiment’s participants even came back to the unfinished tasks after the experiment ended to ultimately finish the task (and they were not asked to do so).

In sum, these classic works from the field of psychology suggest that individuals involved in the execution of high investment tasks, react with (emotional) strain if interrupted. The final completion of the task will remove this strain. These insights mirror in the design of Freemium games such as Candy Crush Saga. This game has been downloaded an incredible 2.7 billion times in five years [84]. In this game, participants must solve levels in which they follow different missions while playing a Tetris like game (although there are obviously several differences between the games, these cannot be presented in detail here). Of note, when the game has not been played for a while, the gamer is endowed with five lives. Some levels are very hard to solve and in case of Candy Crush Saga it is even mentioned that a “super hard level” is coming up. As some of these levels are “super hard” to solve (rumor has it that it is even impossible at first try), players easily loose several of those free lives ending up with no energy to finish this “super hard level”. Being now really attracted by the game, this results in emotional strain which consequently provokes people to spend extra money to buy additional lives/gaming energy, because the next level is only a couple of minutes away.

## 4. Discussion

The present article provided its readers with an overview on often observed elements/features of smartphone/Internet applications in-built to prolong the time spent using a certain app. The depicted elements/features embedded in psychological and economic theory underline the notion that these elements indeed have been designed to prolong app usage. Implementing such elements in a smartphone-app will also contribute to a more addictive nature of apps. From our perspective, it is of tremendous importance to further study such in-built elements, because SUD has been associated with loneliness [85]. Although it is not clear what is hen and what is egg in this work (hence the direction of causality), a growing number of studies indeed support such associations with further demonstrating robust links between SUD and negative emotionality [5]. According to the I-PACE model [26] (and its recent update [86]), a history of psychopathology might indeed be a vulnerability factor to develop IUD (and its mobile form of SUD). Nevertheless, it is also imaginable that withdrawal from society and escapism to the online world ultimately might cause loneliness and reduce social connectedness, too [87]. Additional relevant areas discussed in the literature—probably being a result of too much online consumption—are links between IUD and BMI [88] and between IUD and body image avoidance [89]. The latter effect is likely driven by social comparison processes, in particular when a person is confronted with thin models or other (thin) attractive persons [90]. These effects might be in particular strong on platforms such as Instagram and for those users with low self-esteem [91] and being female [92,93]. In this context also the role of selfies [3,94] and the smartphone camera needs to be mentioned [95].

Of importance, apps on smartphones are not in general bad and not always cause negative emotionality or dissatisfaction with one’s own body image. In contrast, some applications might even foster physical activity and help persons to improve their diet [96]. However, as mentioned, we focused in the present research on the investigation of social media/messenger and Freemium game applications. Such a focus is warranted because several studies already demonstrated that these applications are important drivers of (excessive) smartphone usage (literature on social media/messenger applications and smartphone usage [28,97,98]; literature on gaming and smartphone usage [29,99,100].

Although it needs to be tested further, the here investigated elements of the apps described against psychological and economic theories all likely prolong usage times. Although literature on each of the elements in the study of SUD is scarce, it has been demonstrated that persons have problems in assessing their smartphone consumption, and this is probably due to time distortions [49,101]. Such time distortions are a well-known companion of flow processes as described in 3.1. Beyond the element of flow, a recent study [102] also found empirical support for the endowment effect indeed playing also a role with online products. The authors of this work observed via an online experiment that “consumers become instantaneously attached to and are reluctant to give up digital services once they have obtained them” ([102], p. 311). This all said, the study of elements in-built in smartphone applications present a rather new research endeavor and many questions are still in need to be answered. One of these questions is highlighted in the next section.

The overview so far presented the features separately. In real-world applications, usually several of the explained elements can be found in one application. To our knowledge, studies are largely lacking aiming at an understanding of how strong each of the aforementioned features of the platforms/games impact upon usage time. Clearly, it will be of high interest to know how the different features interact on usage time. For example, when the design of the ‘Newsfeed’ according to the idea ‘show people what they like’ would prolong a person’s stay on Facebook by 10 min, and implementation of the ‘Like’-function by 5 min, will users stay 15 min longer on Facebook or will these effects interact with each other, resulting in 25 min of online app usage? Indeed, this is hard to study for independent scientists because they usually do not get access to data from platforms such as Facebook or Candy Crush Saga. Therefore, one solution will be ultimately to design independent games/social media apps for research purposes where different features are rolled out in different constellations and in different versions of the same game or services. Of note, tech-companies use this approach often and the literature refers here to ‘A/B-testing’. By implementing ‘A/B-testing’, it can easily be tested if version A compared to version B of an online service prolongs usage time. Ethically, it is of great interest, that most of these tests are unsolicited. This also speaks for that ultimately government regulation might be necessary to force the industry to both open up for scientists from different disciplines to be able to study the impact of the aforementioned elements and to build healthier business models.

## 5. Limitations and Changes of the Current Business Model in Apps?

The present work is of theoretical nature and carved out relevant mechanisms likely being designed to prolong usage time of app-services dominating everyday life. Clearly, our assumption that these mechanisms have been purely designed to prolong usage time of an app is speculative, but please see also a larger serious scientific area dealing with persuasive design/technologies in the computer sciences [103,104]. It cannot be said for sure what the app developers had in mind when constructing a certain feature, but some developers, such as Justin Rosenstein, confess about the addictive nature of some of the presented elements [73]. Aside from this, it is very likely that the main intention of implementing features as introduced above in an application, represents the prolongation of app usage, because companies such as Facebook and Google earn their money with data.

Nevertheless, it is mentioned that in the last months a movement has been seen in the Silicon Valley aiming to foster “Time Well Spent” on digital platforms [105]. Applications such as “Screentime” on Apple’s iPhone or “Time Well Spent” on Facebook should help to reduce online time. How good this actually works has not been investigated and please note that some of the authors (together with computer scientists) presented a similar service for Android users already several years ago, hence years before the app industry launched such a laudable initiative (CM and BL as part of a larger development team with their Menthal application [106,107]). However, it seems that even the tech-industry has learned that detrimental effects for mental health can arise due to their developed addictive app mechanisms. Probably implementation of these new control features also reflects a reaction to pressure from regulating bodies. Results from studies investigating the effect of smartphones and/or social media use on well-being came up with diverse results [28,108,109,110]. Hence, it also needs to be better understood what kind of use-reduction goes along with the healthiest effects.

Although no one can really predict if this is going to happen soon, the future will see if other healthier business models will be built into applications without the addictive potential currently seen in many of the prominent and often used applications. Apart from these new developments, the recent work by Alutaybi et al. [45] needs to be mentioned, because it came up with ideas on how to reduce FoMO and thereby likely also to reduce addictive tendencies towards social media applications. Before going a bit more in detail concerning some of these ideas, it is noteworthy that the authors presented FoMO eliciting features such as ‘temporal events’ in their article going beyond what is presented in our work, perhaps also resulting in more addictive tendencies. Of note, a ‘temporal event’ could be defined by an information being available only for a short time period (this was also one of the most prominent Snapchat functions): Either a person is then fast in consuming the content or simply has not seen what was going on. The authors end their interesting article with thoughts on “FoMO-Aware SNSs Design” [45] (p. 4), in which they basically think about designing social media platforms giving users the ability to make a short note on when they are available. This could help in reducing the urge to constantly check social media, because others are aware of a person being absent (and therefore not being able to reply), but also to set up bilateral or even collective protocols providing persons with rules on when they want to respond to each other. The effects of such an intervention clearly still need to be empirically tested. When comparing the work by Alutaybi et al. [45] and the present one, both of them have merits, as the former has more of a computer science design approach to its analysis (also in carving out ‘addictive’ or FoMO elements), whereas in the present work it is stronger focused on psychological/economic theories as described above.

Finally, the prominent honeycomb model by Kietzmann et al. [111] is mentioned, because it represents one of the first models to systematically analyze the diverse building blocks of social media. Of importance, this early very influential work addressed business persons in order to help them to recognize the most congruent social media platform for their companies in terms of a social media platform’s building blocks called identity, conversation, sharing, presence, relationships, reputation, and groups. When focusing solely on addictive tendencies of social media sites, this framework might be fruitful to be brought together with the present psychological theories in order to empirically tackle the question in which area or areas of the honeycomb model, which of our presented features might have what kind of impact on usage time.

## Figures and Tables

**Table 1:** Elements used to prolong usage time of social media apps and/or Freemium games.

Psychological Mechanisms Built-in Social Media/Messenger Apps and/or Freemium Games	Example/Illustration
Endless scrolling/streaming	As soon as one video is at the end on a website such as YouTube, the next video begins with either a similar content or the second episode of a TV show and so forth. By this, viewers get more and more absorbed, which makes it hard to stop watching.
Endowment effect/ mere-exposure effect	Every time players visit the app platform and invest more time in the construction of the virtual world, it will get harder for them to detach from the game or even delete the app. The endowment effect might be both explained by ownership and loss aversion. Also, of importance is the mere exposure effect describing that the more often you are exposed to a certain (neutral) thing or application (here a game), the more you like it.
Social pressure	Illustration from a WhatsApp feature: If a user sends a message to a friend, the sender is presented with two gray ticks, which means that the message has successfully arrived at the recipient’s phone. If the recipient reads the message, the grey ticks turn blue. As both sides know about these rules, social pressure emerges. Both parties likely expect a fast answer, above all, if the message apparently has been read.
Show users of an app what they like	Facebook has a great interest in studying the behavior of each person at perfection and in much detail, so that at best only such information is presented in the ‘Newsfeed’ which is most interesting for the user. Otherwise, people could get bored and close the browser window.
Social comparison and social reward	Perhaps one of the most prominent features of social reward mechanisms in social media is the iconic ‘thumbs up’. A ‘thumbs up’ (‘Like’) demonstrates either positive social feedback on one’s own post or gives another person such a feedback.
Zeĭgarnik effect/ Ovsiankina effect	The Zeigarnik effect refers to better remembering of tasks, where a person has been interrupted. Rickers-Ovsiankina then showed that such interrupted tasks are more likely to be finished later on (even if one is not forced to do this). Illustration: Some levels in Freemium games are very hard to solve and in case of Candy Crush Saga it is even mentioned that a “super hard level” is coming up. As some of these levels are “super hard” to solve (rumor has it that it is even impossible at first try), players easily loose several of those free lives ending up with no energy to finish this “super hard level”. Being now really attracted by the game, this results in emotional strain which consequently provokes people to spend extra money to buy additional lives/gaming energy, because the next level is only a couple of minutes away.
